# Rod bipolar cells dysfunction occurs before ganglion cells loss in excitotoxin-damaged mouse retina

**DOI:** 10.1038/s41419-019-2140-x

**Published:** 2019-12-02

**Authors:** Yumeng Shen, Xue Luo, Shiliang Liu, Ying Shen, Scott Nawy, Yin Shen

**Affiliations:** 1 Eye Center, Renmin Hospital of Wuhan University, Wuhan University, Wuhan, 430060 Hubei Province China; 2 Department of Ophthalmology, Tongji Hospital, Tongji Medical College, Huazhong University of Science and Technology, 1095 Jiefang Avenue, Wuhan, 430030 Hubei Province China; 3 Medical School, Zhejiang University, Hangzhou, 310053 Zhejiang Province China; 4 Department of Molecular and Cell Biology, University of California Berkeley, Berkeley, 94720 CA USA

**Keywords:** Cell death in the nervous system, Diseases of the nervous system

## Abstract

Progressive degeneration of retinal ganglion cells (RGCs) will cause a blinding disease. Most of the study is focusing on the RGCs itself. In this study, we demonstrate a decline of the presynaptic rod bipolar cells (RBCs) response precedes RGCs loss and a decrease of protein kinase Cα (PKCα) protein expression in RBCs dendrites, using whole-cell voltage-clamp, electroretinography (ERG) measurements, immunostaining and co-immunoprecipitation. We present evidence showing that N-methyl D-aspartate receptor subtype 2B (NR2B)/protein interacting with C kinase 1 (PICK1)-dependent degradation of PKCα protein in RBCs contributes to RBCs functional loss. Mechanistically, NR2B forms a complex with PKCα and PICK1 to promote the degradation of PKCα in a phosphorylation- and proteasome-dependent manner. Similar deficits in PKCα expression and response sensitivity were observed in acute ocular hypertension and optic never crush models. In conclusion, we find that three separate experimental models of neurodegeneration, often used to specifically target RGCs, disrupt RBCs function prior to the loss of RGCs. Our findings provide useful information for developing new diagnostic tools and treatments for retinal ganglion cells degeneration disease.

## Introduction

Retinal bipolar cells, receive light signal inputs from rods or cones, and send outputs to ganglion and amacrine cells^1^. Differences in the glutamate subtypes expressed in different classes of bipolar cells underlie the fundamental segregation of visual information into two parallel pathways: the ON and OFF pathway, which differ in the polarity of their light response. In particular, the ON pathway is mediated by rod bipolar cells (RBCs) which receive input exclusively from rods, and ON cone bipolar cells (ON-CBCs) which collect input from cones, both respond to light increments with membrane depolarization. The visual signal transduction machineries in RBCs and ON CBCs have significant similarities, as their signals are both mediated by the metabotropic glutamate receptor metabotropic glutamate receptor 6 (mGluR6)^2^ and the transient receptor potential cation channel subfamily M member 1 (TRPM1) channel^3^. However, some key cellular regulators, such as PKCα have been shown to be specifically expressed in RBCs, but not in ON CBCs^4^.

PKCα is a calcium-dependent protein kinase, has been implicated in various pathological and physiological processes^5^. Since its activation requires an increase of intracellular free Ca^2+^ and binding to diacylglycerol (DAG). Activation of PKCα induces a conformational change and translocation to the cell membrane^6^. In the retina, PKCα regulates GABAergic feedback onto RBC terminal, reducing GABA-induced currents generated by the release of GABA from amacrine cells^7–9^. PKCα has also been suggested to play an important role in the modulation of TRPM1^10–12^, the transduction channel that mediates postsynaptic currents in RBCs. However, the exact function of PKCα in RBCs remains elusive.

Activation of PKCα increases its binding with PICK1^13^ and PICK1 is phosphorylated following activation of Ca^2+^/CaMKII (Ca^2+^/calmodulin-dependent protein kinase II)^14,15^. PKCα can be degraded by the ubiquitin-proteasome system (UPS), a major intracellular protein degradation pathway^16–18^.

Here we show that three distinct models of retinal disease, that have been traditionally used to target RGCs dramatically reduce PKCα levels in RBCs. Remarkably, the reduction in PKCα expression was correlated with a functional loss of synaptic responsiveness, and both events occurred at time points prior to RGCs loss in all three models. Responsiveness was partially recovered by strategies that unregulated PKCα, and was completely recovered in a mouse in which the PKC interacting protein PICK1 was knocked out. Modulation of PKCα could be a potential novel mechanism for regulating signal transduction and visual processing in the retina rod pathway.

## Results

### PKCα in RBCs is degraded in retina neurodegeneration models

**Fig. 1:**
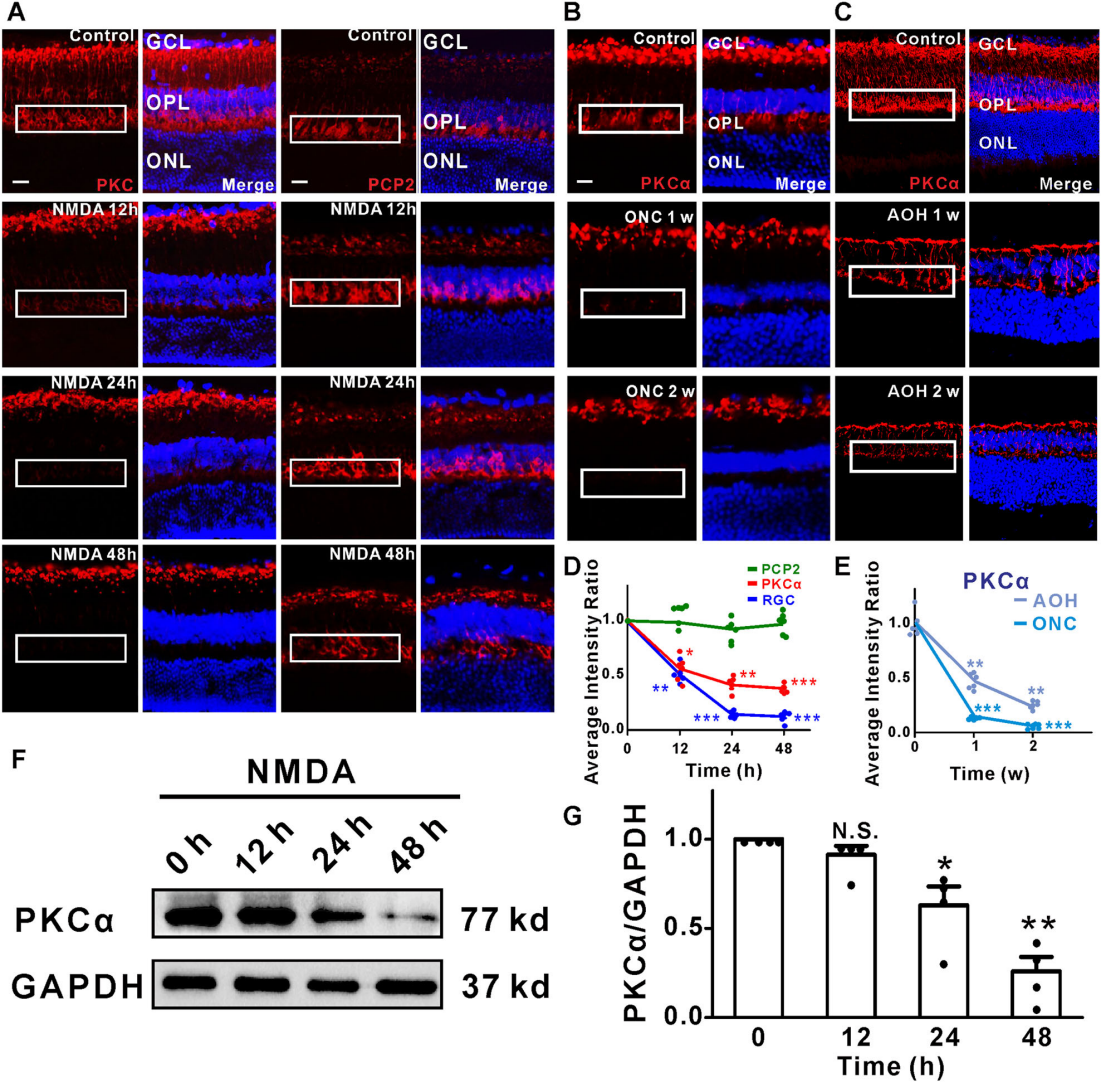
PKCα degradation in RBCs dendrites appeared to be a universal mechanism for RGCs loss in experimental glaucoma models. **a** PKCα expression at the somas and dendrites of RBCs was reduced dramatically after intravitreal injection of NMDA (left), while PCP2 expression remained the same with NMDA treatment (right). **b** Decreased PKCα expression in RBCs dendrites induced by optic nerve crush (ONC) model. **c** Representative images of PKCα (red) of retinas treated with the acute ocular hypertension (AOH) glaucoma model. Decreased PKCα expression in RBCs dendrites, and a reduction of the thickness of the retina at time points following administration of NMDA. **d**, **e** Fluorescence intensity of PKCα, PCP2, and DAPI in OPL decreased after NMDA treatment. PKCα average intensity ratio of NMDA 12, 24, and 48 h, *n* = 5: 0.56 ± 0.06, *P* = 0.0149; 0.41 ± 0.04, *P* = 0.0013; 0.38 ± 0.02, *P* = 0.0002. **f**, **g** The PKCα protein expression level decreased significantly after the administration of NMDA. Average intensity of PKCα/GAPDH of NMDA 12, 24, and 48 h, *n* = 4: 0.91 ± 0.05, *P* = 0.1822; 0.63 ± 0.11, *P* = 0.0396; 0.26 ± 0.08, *P* = 0.0029. INL, inner nuclear layer; GCL, ganglion cell layer; OPL: outer plexiform layer. Scale bar = 50 μm.

Glaucoma is associated with progressive loss of RGCs and optic nerve damage. Although the endpoint is RGC death, the underlying etiologies are varied, it is useful to characterize damage not only to RGCs, but other retinal cells, using methodologically distinct models. Here we used several established mouse models of RGC injury, including acute intravitreal injection of NMDA, optic nerve crush (ONC), and acute ocular hypertension (AOH)^19^. At 12, 24, and 48 h time points following NMDA injection, the expression of PKCα, an important cellular marker and regulator of RBCs^11,20^ was significantly decreased in RBCs and their dendrites, compared to a sham injected control group (Fig. 1a, d). The result was also consistent with the PKCα protein expression level decrease in Fig. 1f, g. Intravitreal injection of NMDA at a concentration of 10 mM effectively eliminated RGCs and their associated optic nerve axon in a time-dependent manner (Supplementary Fig. 1), as shown by the previous studies^5,21–23^. In the anterior chamber perfusion pressure model, which is an acute high IOP model, the expression of PKCα, decreased progressively in the RBCs cell bodies and dendrites after acute IOP elevation (Fig. 1c). Moreover, in the ONC model, which is a model of RGC degeneration that is unrelated to changes in intraocular pressure (IOP)^24^, PKCα significantly decreased within 1–2 weeks (Fig. 1b). Although bipolar cells are interneuron whose axons do not project into the optic nerve, damage to this nerve none-the-less resulted in significant loss of PKCα. Thus, PKCα degradation in RBC dendrites is a common hallmark of three different retinal injury models.

To test if PKCα protein reduction was due to the loss of RBCs, we monitored the expression of another well-established RBCs marker, Purkinje cell protein 2 (PCP2)^25^. PCP2 remained unchanged in RBCs from both control and NMDA-treated retinas (Fig. 1a), suggesting that the loss of PKCα expression after NMDA treatment is not due to RBC loss.

### NMDA disrupts the function of RBCs, but not CBCs

**Fig. 2:**
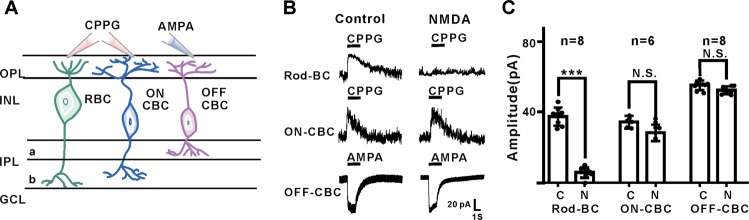
NMDA treatment selectively decreased the function of rod bipolar cells but not cone bipolar cells. **a** Sketch of patch clamp of retina bipolar cells. **b** NMDA dramatically decreased the CPPG-evoked response (100 μM, V_hold_ = +40 mV) of RBCs, whereas injected NMDA did not affect the CPPG-evoked response (100 μM, V_hold_ = +40 mV) of ON-CBC or the AMPA-evoked response (100 μM, V_hold_ = −40 mV) of OFF-BC significantly. **c** Quantification of NMDA treatment on CPPG-evoked/AMPA-evoked responses in different bipolar cells. OFF BC mean amplitude = 55.08 ± 0.91 pA, NMDA treatment mean amplitude = 52.26 ± 0.78 pA, *P* = 0.063; ON-cone BC mean amplitude = 33.11 ± 1.26 pA, NMDA treatment mean amplitude = 29.83 ± 1.77 pA. *n* = 6; *P* = 0.2491; RBC mean amplitude = 37.38 ± 1.82 pA, NMDA treatment mean amplitude = 5.88 ± 1.03 pA; *n* = 8; *P* < 0.0001.

As NMDA decreased PKCα expression in RBCs, we wondered whether this morphological change would, in turn, alter the function of RBCs. We performed patch-clamp recordings of RBCs, cone ON bipolar cells (CBCs) and OFF bipolar cells, which exhibit distinctive patterns of axon terminals in the inner plexiform layer (IPL) and different light responses. Application of (RS)-α-cyclopropyl-4-phosphonophenylglycine (CPPG) in the presence of the mGluR6 agonist L-AP4 has been used as an effective proxy for light-evoked responses in RBCs^9,26–28^, and AMPA for light-evoked responses in Off bipolar cells^29,30^. In the control retina, ON bipolar cells (including RBCs and ON-CBCs) were depolarized by puffs of the mGluR antagonist CPPG (100 μM, V_hold_ = +40 mV) in the presence of L-AP4 (4 μM), whereas OFF bipolar cells were depolarized by puffs of AMPA (100 μM, V_hold_ = −40 mV). After NMDA treatment, there was no significant change in the amplitudes of CPPG-evoked responses in ON-CBCs or OFF bipolar cells, in sharp contrast to the significantly decreased amplitude in RBCs (Fig. 2b). These results demonstrated that NMDA treatment disrupted the function of RBCs, but not CBCs.

**Fig. 3:**
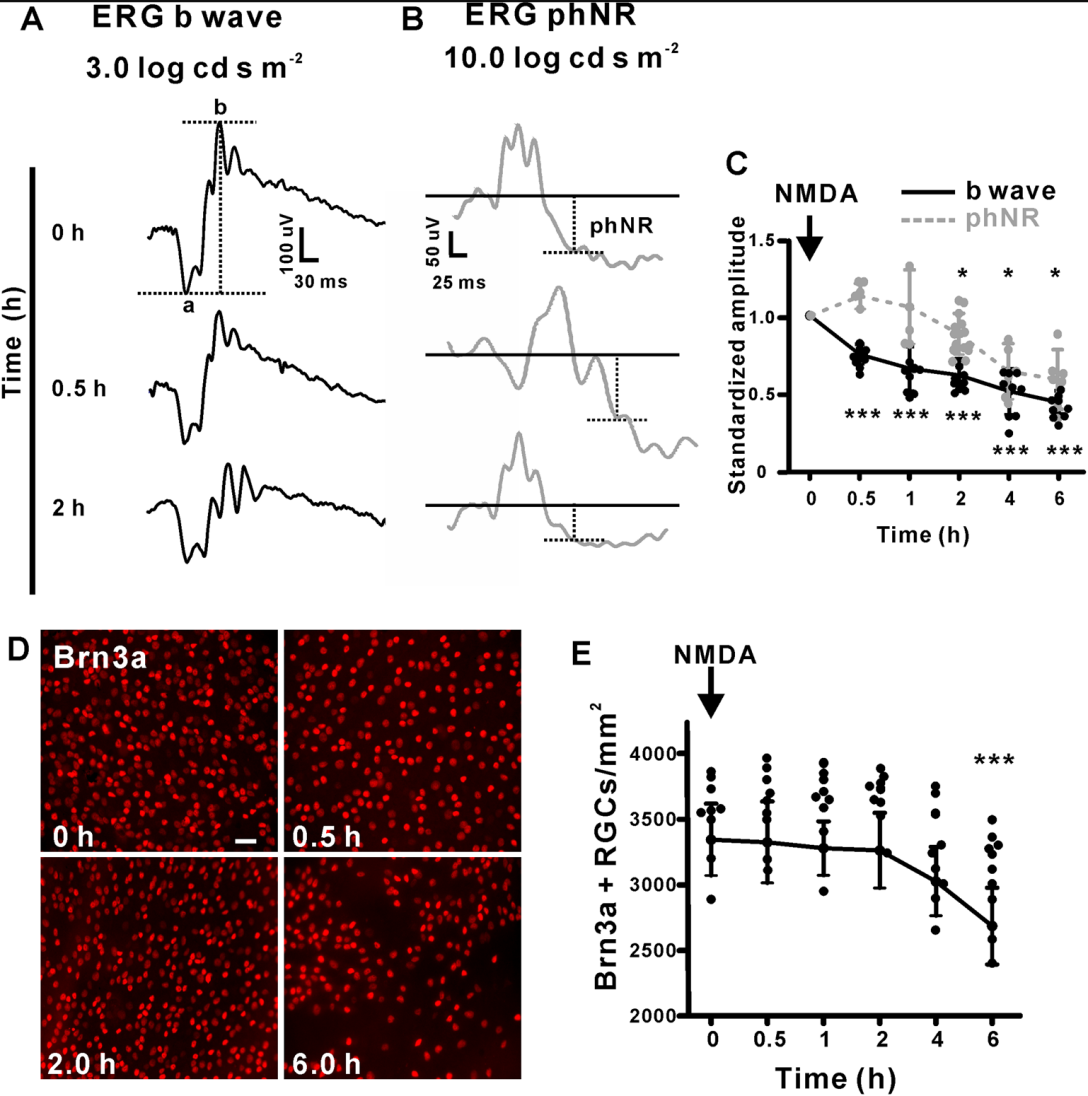
Functional deficits in RBCs appears prior to RGC loss induced by NMDA. **a** Representative scotopic ERG waves at different times after NMDA treatment. **b** Representative photopic ERG waves after 2 h of NMDA treatment. **c** Quantification of the b wave and PhNR amplitude at different time points of treatment. The standardized b-wave and PhNR of after injection of NMDA at the indicated times. 0.5, 1.0, 2.0, 4.0, and 6.0 h group, *n* = 9: 0.75 ± 0.06, *P* < 0.0001; 0.66 ± 0.14, *P* < 0.0001; 0.61 ± 0.11, *P* < 0.0001; 0.51 ± 0.15, *P* < 0.0001; 0.43 ± 0.08, *P* < 0.0001. **d** Example images of retinal flatmount preparations labeled with Brn3a (red). The number of labeled RGCs decreased significantly when treated with 0.05 mM NMDA for 6 h. (scale bar = 50 μm). **e** Quantification of the numbers of RGCs/mm^2^. The RGCs number of NMDA 0.5 h, 1.0, 2.0, 4.0, and 6.0 h group, *n* = 9: 3326.48 ± 311.43, *P* = 0.65; 3280.18 ± 205.39, *P* = 0.51; 3264.32 ± 287.20, *P* = 0.72; 3028.12 ± 262.96, *P* = 0.07; 2686.90 ± 292.91, *P* = 0.0002.

To compare the timing of RGC loss with the reduction in RBC signaling, we measured the amplitude of the scotopic ERG b-wave, which represents the function of ON bipolar cells, together with the PhNR wave, which represents the function of RGCs. After treatment with a low concentration of NMDA (0.05 mM) for 0.5 h, the b-wave decreased significantly (Fig. 3a, c), while the ERG PhNR wave decreased after 4 h, concomitant with the appearance of RGC loss (Fig. 3d, e). This indicates that RBCs are functionally compromised prior to RGCs in the NMDA model.

### RBCs function impairment through reduced PKCα expression

**Fig. 4:**
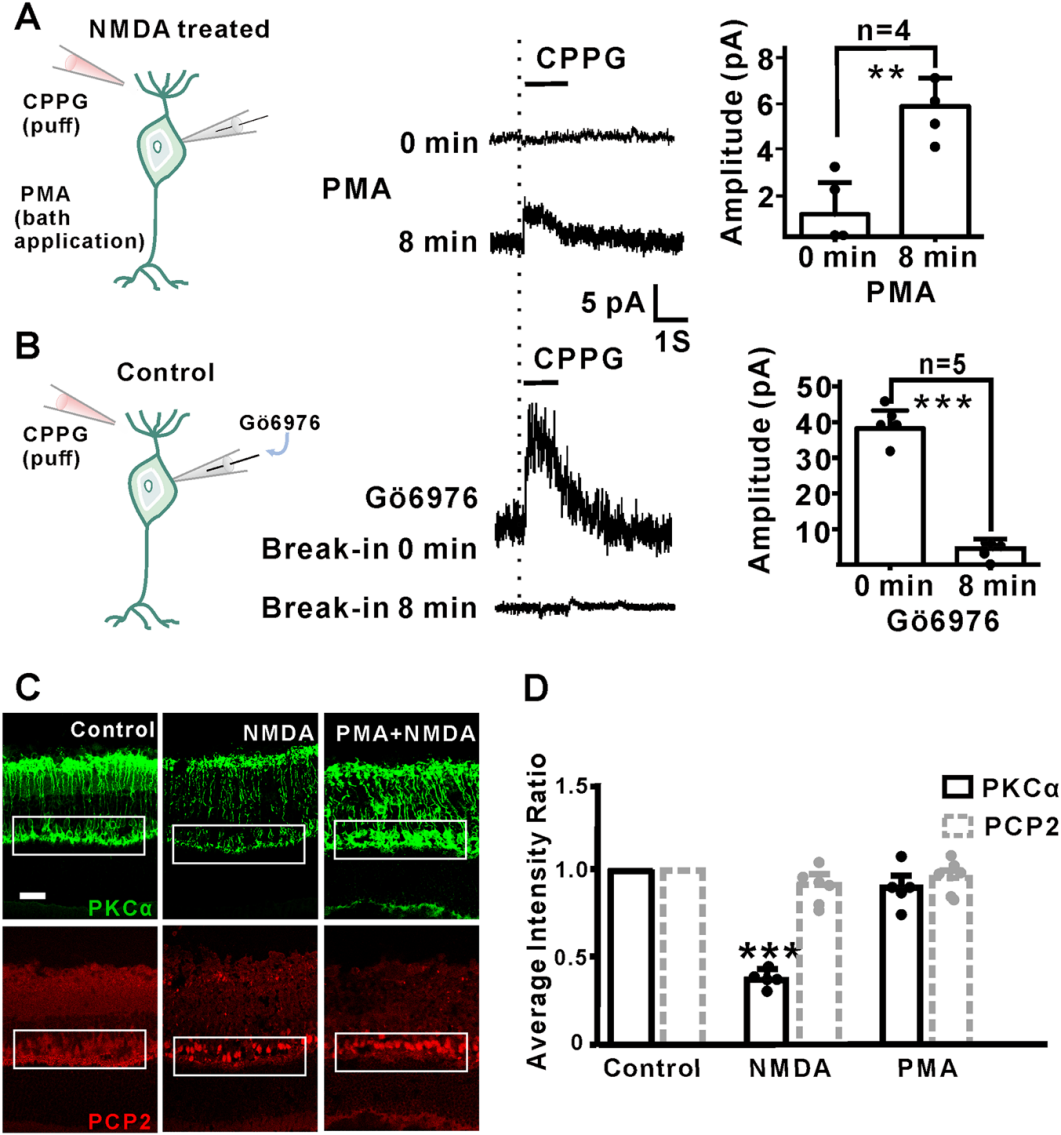
PKCα is essential for RBC activity. **a** In retinal patch-clamp recording, CPPG failed to induce an RBC response in NMDA-treated retina (gray line); but RBC responses could be rescued after bath applying the PKCα activator PMA for 8 min (dark line). NMDA group, mean amplitude = 1.00 ± 1.41 pA, NMDA + PMA group, mean amplitude = 5.80 ± 1.30 pA, *P* = 0.0093. **b** Dialysis of the PKCα inhibitor Gö6976 in the recording peptide completely blocked the RBC transduction response after 8 min break-in (dark line) in whole-cell recording. 0 min of recording; mean amplitude = 39.14 ± 5.05 pA, 8 min of recording; mean amplitude = 3.80 ± 2.39 pA, *P* < 0.0001. **c** PKCα staining at the somas and dendrites was reduced dramatically after the intravitreal injection of NMDA. PKCα activation (PMA) could rescue PKCα loss at dendrites without changing PCP2 (red) expression (scale bar = 50 μm). **d** Quantification average fluorescence intensity of PKCα and PCP2. PKCα average intensity ratio of NMDA 24 h compared to control: *n* = 5: 0.38 ± 0.05, *P* = 0.0005, and PMA + NMDA vs. control: 0.92 ± 0.15, *P* = 0.4214;PCP2 average intensity ratio of NMDA 24 h vs. control: *n* = 5: 0.90 ± 0.04, *P* = 0.1941; PMA + NMDA vs. control: 0.97 ± 0.04, *P* = 0.6878.

To further investigate how PKCα reduction influenced the function of RBCs, patch-clamp recording was used to examine the CPPG-induced responses in control and NMDA-treated retinal slices in the presence of PKCα inhibitors or activators. Strikingly, the diminished CPPG-evoked transduction response in NMDA-treated RBCs was partially rescued by the PKCα activator PMA (Fig. 4a). Conversely, incubation of the retina with the PKCα inhibitor GÖ6976 completely blocked the CPPG-induced transduction response in RBCs (Fig. 4b). PMA treatment also rescued the expression of PKCα as shown by immunofluorescence staining (Fig. 4c). Taken together, these results suggest that PKCα is a critical downstream effecter of NMDA, and that NMDA-induced downregulation of PKCα disrupts the normal function of RBCs.

### Involvement of PICK1 and phosphorylated NR2B in PKCα degradation

**Fig. 5:**
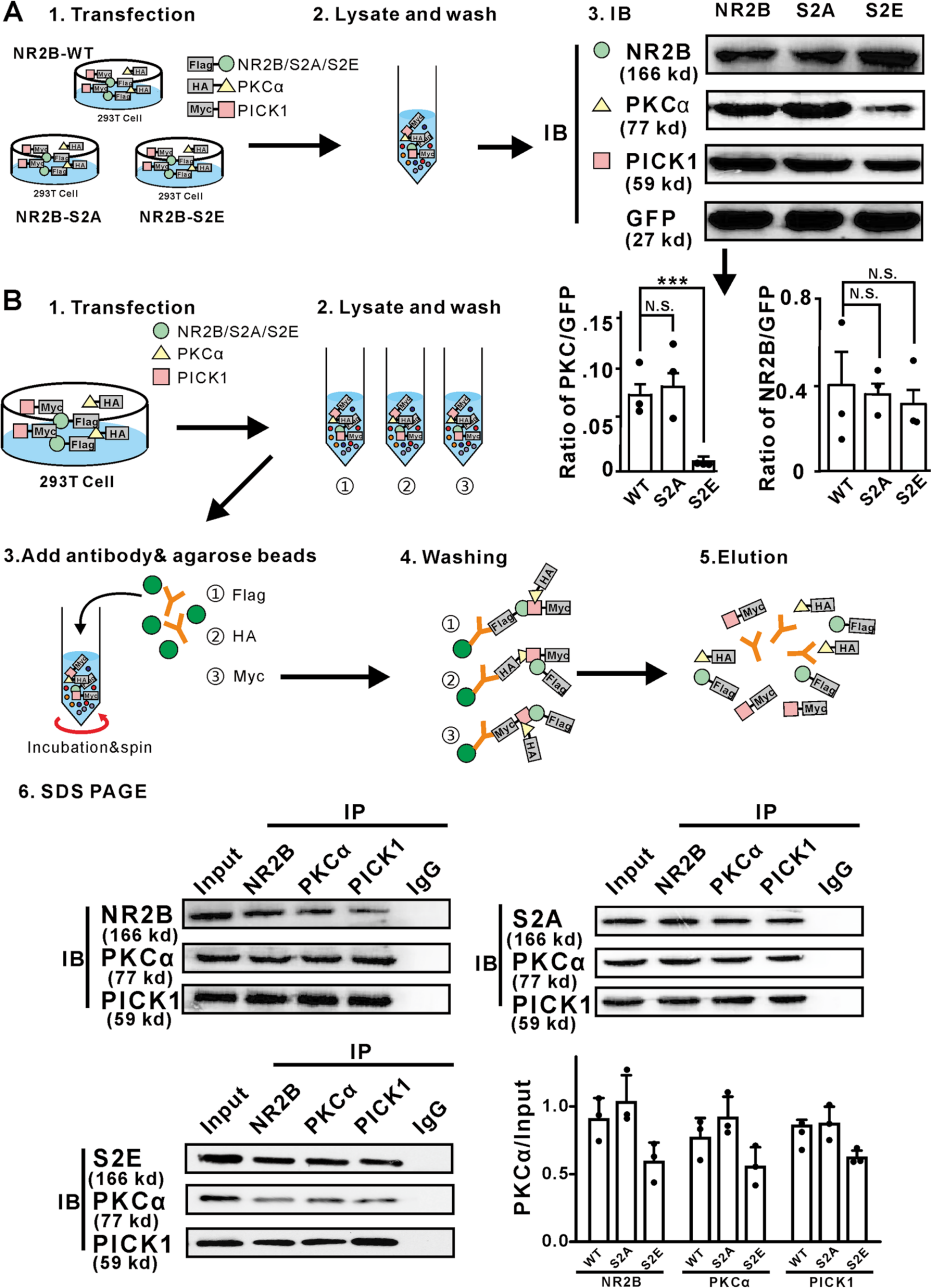
NR2B interacted with PKCα through PICK1, phosphorylated NR2B leaded to promote PKCα degradation. **a** HEK293T cell lysates co-transfected of NR2B (or NR2B-S2A, NR2B-S2E), PKCα, PICK1, and GFP plasmids were immunoblotted. The expression of PKCα decreased significantly when co-transfected with PICK1 and NR2B-S2E (**). The expression intensity of NR2B compared to GFP was not significantly different among three groups. The expression intensity of PKCα compared to GFP decreased significantly in NR2B-S2E group. Average intensity of PKCα/GFP: NR2B, 0.08 ± 0.01; NR2B-S2A, 0.09 ± 0.02, *P* = 0.1046, NR2B-S2E, 0.008 ± 0.0003, *P* = 0.0007. NR2B-S2A: The non-phosphorylated form of NR2B; NR2B-S2E: phosphorylated-mimicking form of NR2B. **b** HEK293T cell lysates co-transfected of NR2B (or NR2B-S2A, NR2B-S2E), PKCα and PICK1 plasmids were immunoprecipitated (IP) and immunoblotted (IB). The control was incubated with IgG antibody. NR2B, PKCα and PICK1 combined together as a triad complex. Average intensity of PKCα/Input of WT, S2A, and S2E group, *n* = 3: NR2B: 0.90 ± 0.16; 1.03 ± 0.20, *P* = 0.5958; 0.5958pnt, *P* = 0.2100; S2A: 0.77 ± 0.15, *P* = 0.1606; 0.9106776, *P* = 0.1606, *P* = 0.4933; 0.55 ± 0.15, *P* = 0.4933; S2E: 0.86:4933; 0.87 ± 0.13, *P* = 0.5732; 0.62 ± 0.06, *P* = 0.09.

The functional properties of NMDA receptors have previously shown to be markedly influenced by incorporation of the regulatory subunit NR2B and are important for PKCα trafficking in neuronal cells^31,32^. Phosphorylation of synthetic peptides have indicated that the Ser1303 site on NR2B is a PKCα substrate in vitro and that PKCα can directly phosphorylate Ser1303, leading to enhanced calcium influx through NMDA receptor channels^33^. PICK1 is also a PKCα binding protein essential for synaptic plasticity^34^. As NR2B and PICK1 were both functionally connected to PKCα in previous studies, we speculated that these three proteins may physically interact with each other to form a complex in retinal RBCs. When NR2B, PICK1, and PKCα were co-expressed, only NR2B-S2E (phosphorylation-mimicking derivative) but not NR2B (WT) or NR2B-S2A (non-phosphorylatable derivative) promoted the degradation of PKCα (Fig. 5a), suggesting that the phosphorylated form of NR2B leads to PKCα degradation. Moreover, consistent with previous studies, NR2B, PKCα, and PICK1 could be co-immunoprecipitated after co-expression in 293T cells, but the binding efficiency had no significant difference (Fig. 5b). Thus, phosphorylated NR2B promotes the degradation of PKCα by binding with it in a PICK1-dependent manner, forming a NR2B–PICK1–PKCα complex.

### Enhanced NR2B phosphorylation is CaMKII-dependent

**Fig. 6:**
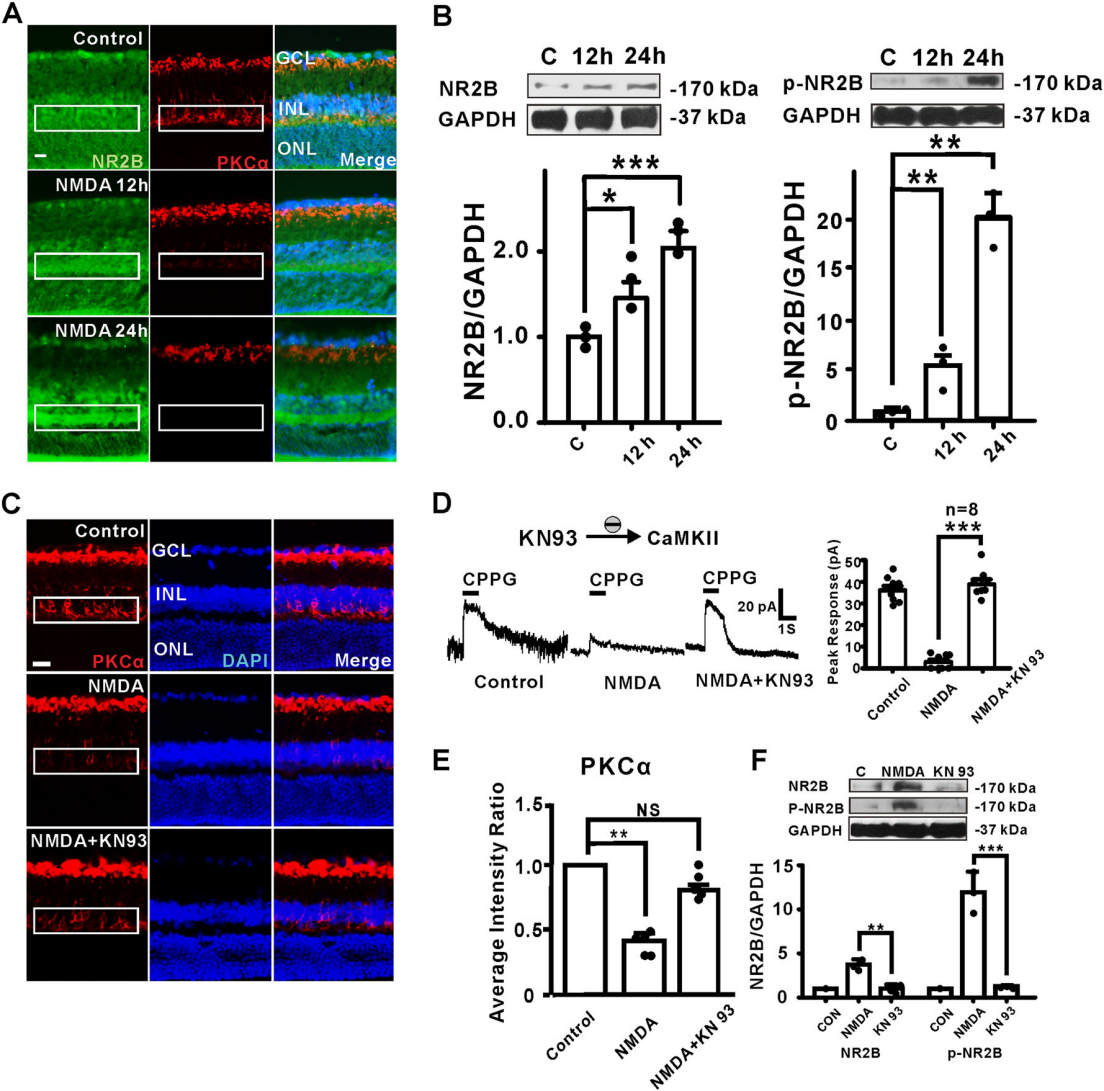
NMDA treatment increased NR2B protein expression and its CaMKII-dependent phosphorylation levels in RBCs. **a** NMDA-induced increased expression of NR2B was markedly decreased by KN93 treatment. The rod bipolar cell dendrites, axons, and axon terminals are NR2B-positive. PKCα staining in the dendrites and somas decreased, whereas NR2B staining in the same layer significantly increased after NMDA treatment (scale bar = 50 μm). **b** Expression of NR2B and p-NR2B increased in a time-dependent manner (target molecule/GAPDH). Average intensity ratio of NR2B/GAPDH: NMDA 12 h = 1.46 ± 0.18, *P* = 0.0499; NMDA 24 h = 2.04 ± 0.20, *P* = 0.0009. **c**, **e** Representative images of PKCα (red) Immunostaining after intravitreal injection of vehicle, NMDA or NMDA + KN93. PKCα average intensity ratio of NMDA 24 h vs. KN93 + NMDA, *n* = 5: 0.41 ± 0.04, *P* = 0.0013; 0.87 ± 0.05, *P* = 0.119 (scale bar = 50 μm). **d** Co-treatment of KN93 significantly reversed the decreased RBC response compared to the NMDA-treated retina. Mean amplitude: control group, 36.07 ± 2.08 pA, NMDA group, 3.0 ± 1.15 pA, NMDA + KN93 group, 38.93 ± 2.21 pA. *n* = 8, *P* < 0.0001. **f** NMDA-induced increased expression of NR2B was markedly decreased by KN93 treatment. Average intensity of NR2B/GAPDH: NR2B, NMDA 24 h = 3.75 ± 0.60, NMDA + KN93 = 1.01 ± 0.48, *P* = 0.0072; p-NR2B, NMDA 24 h = 11.96 ± 2.31, NMDA + KN93 = 1.29 ± 0.12, *P* = 0.0007.

A dramatic time-dependent increase of the NR2B subunit, and a concomitant decrease in expression of PKCα in RBCs dendrites was observed following intravitreal injection of NMDA (Fig. 6a, b). Besides NR2B, the NMDA receptor subunits NR1 and NR2D both showed elevated expression in RBCs of the NMDA-treated retina as well (Supplementary Fig. 2). As NR2B is a known substrate of Ca^2+^/calmodulin-dependent protein kinase II (CaMKII), we examined the potential role of CaMKII in the NMDA-induced PKCα degradation. The protein levels of NR2B and phosphorylated NR2B were significantly higher in the NMDA-treated retina compared to those in the untreated retina (Fig. 6b). Remarkably, when the administration of NMDA was combined with KN93, a CaMKII inhibitor, the NMDA-induced PKCα degradation was largely reversed (Fig. 6c, e). Since NMDA receptor channel blocker MK801 but not memantine (a parasynaptic NMDA receptor antagonist), could reverse the PKCα decrease in RBCs due to NMDA treatment (Supplementary Fig. 3). Moreover, KN93 completely reversed the inhibitory effect of intravitreal injection of NMDA on the CPPG-evoked responses in RBCs (Fig. 6d), and KN93 substantially reduced levels of total NR2B and phosphorylated NR2B (Fig. 6f). Taken together, these results suggest that Ca^2+^/CaMKII play a critical role in promoting NR2B-mediated PKCα degradation.

### PICK1 was indispensable for NMDA-induced PKCα degration

**Fig. 7:**
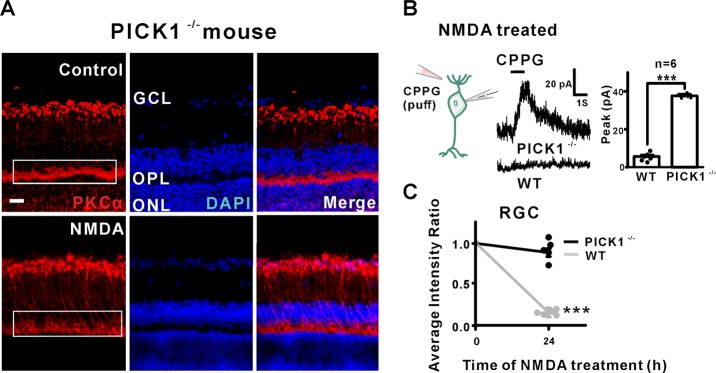
PICK1 was required for NMDA-induced PKCα protein maintain in RBCs dendrites. **a** In PICK1 knockout mouse, PKCα expression (red) is unchanged following NMDA treatment (scale bar = 50 μm). **b** Example of the response of RBCs to CPPG following treatment with NMDA in PICK1 knockout wild type mouse. WT mean amplitude = 3.5 ± 1.27 pA, PICK1^−/−^ mean amplitude = 38.93 ± 2.21 pA, *n* = 6, *P* < 0.0001. **c** Summary data showing that the expression of RGCs decreased dramatically in WT mouse with NMDA treatment but not in PICK1 knockout mouse. DAPI average intensity ratio of PICK1^−/−^ + NMDA = 0.91 ± 0.05, *n* = 5, *P* = 0.4948.

As PICK1 is both an interacting partner of PKCα and a phosphorylation substrate of Ca^2+^/CaMKII^13–15^, we explored the possible role of PICK1 in Ca^2+^/CaMKII-dependent degradation of PKCα in RBCs. First, we compared retinas from PICK1^−/−^ mice with or without NMDA treatment. In sharp contrast to the results from wide-type mice (Fig. 1), no significant change of PKCα expression was observed in RBCs of PICK1^−/−^ mice (Fig. 7a). Furthermore, there was no decrease in RGCs number (Fig. 7c). Consistent with this finding, the CPPG-evoked response was not affected in PICK1^−/−^ retina after NMDA treatment, in contrast to the diminished response observed in the WT retina (Fig. 7b). In conclusion, our results implicate PICK1 as an essential element for NMDA-induced PKCα degradation and RBCs dysfunction.

### The ubiquitin-proteasome system mediated NMDA-induced PKCα degradation

**Fig. 8:**
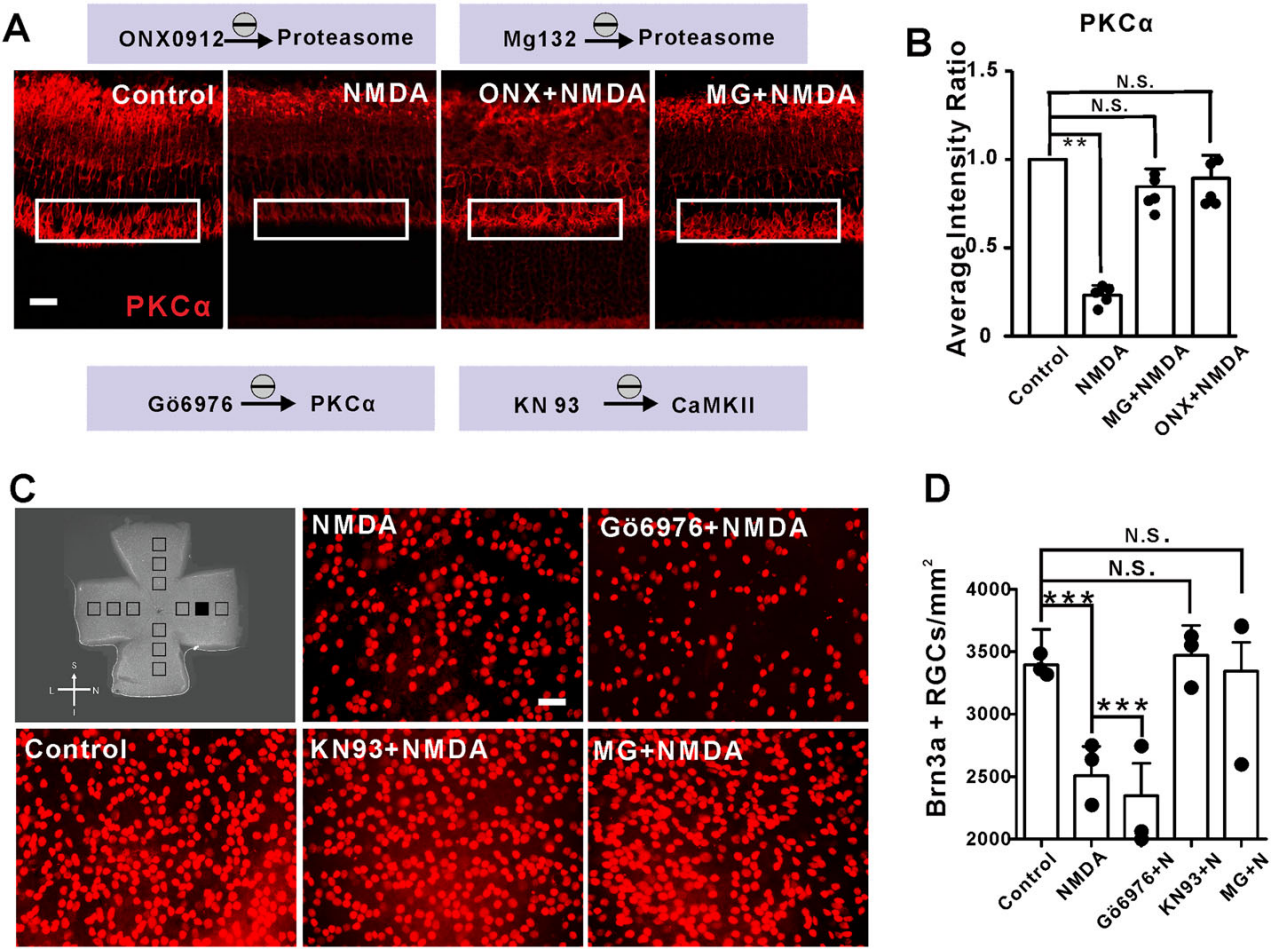
NMDA-induced PKCα degradation in RBCs dendrites was ubiquitin-proteasome-dependent. **a**, **b** Representative images of PKCα (red) after intravitreal injection of vehicle, NMDA, ONX0912 + NMDA and MG132 + NMDA. (ONX0912, MG132: ubiquitin-proteasome inhibitor). ONX0912 and MG132 could rescue decreased expression of PKCα induced by NMDA, but could not rescue RGCs apoptosis. PKCα average intensity ratio of MG132 + NMDA and ONX0912 + NMDA, *n* = 5: 0.85 ± 0.04, *P* = 0.1348; 0.89 ± 0.06, *P* = 0.3795 (scale bar = 50 μm). **c**, **d** Representative retinal flatmount preparations stained with Brn3a. Images were obtained from the region corresponding to the filled box in (**c**). All Brn3a + RGCs in boxes were counted for (**d**). KN93 and MG132 could reverse the RGCs loss caused by NMDA. However, Gö6976 exacerbated the RGCs loss. RGCs/mm^2^: Control = 3396.205 ± 282.180; NMDA = 2507.145 ± 236, *P* < 0.0001; Gö6976 + NMDA = 2347.108 ± 260.037, *P* < 0.0001; KN93 + NMDA = 3471 ± 239.763, *P* = 0.1492; MG132 + NMDA = 3343.621 ± 232, *P* = 0.7762.

PKCα degradation has been suggested to be mediated by the ubiquitin-proteasome system (UPS)^16–18^. The proteasome inhibitors MG132 and ONX0912 were used to test the possibility of UPS mediated PKCα degradation in the NMDA model of neurodegeneration. Consistently, both MG132 and ONX0912 prohibited NMDA-induced PKCα degradation in bipolar cell dendrites (Fig. 8a, b), indicating that PKCα was degraded by the ubiquitin-proteasome system in RBCs when treated with NMDA.

Interestingly, the molecular mechanisms governing the downregulation of RBC function paralleled the NMDA-dependent loss of RGCs. MG132 dramatically reduced RGCs loss caused by NMDA, as did the Ca^2+^/CaMKII inhibitor KN93 (Fig. 8c, d). Furthermore, the PKCα inhibitor GÖ6976 exacerbated NMDA-mediated excitotoxic damage to RGCs (Fig. 8c, d). It remains to be determined if NMDA acts independently to trigger these events in RGCs and RBCs, or whether the two events are causally related to each other.

## Discussion

Retinal bipolar cells, which send visual signal to RGCs, have been largely overlooked in previous studies of glaucoma. In humans, several clinical studies have reported scotopic ERG changes in advanced glaucoma^35–37^. Here, we show that RBCs are functionally affected as early, or earlier than RGCs, and provide evidence that the early dysfunction of RBCs is associated with the degradation of PKCα in RBCs, possible through the complex of NR2B–PICK1–PKC dependent ubiquitin-proteasome system.

Glaucoma has traditionally been considered a disease that is limited to the loss of RGCs. However, recent studies suggest that retinal pathology in glaucoma may involve retinal amacrine cells^38–40^. Here we provide evidence that the cellular function of RBCs was disrupted in several mouse models, of neurodegeneration, as the function of RBCs (presented as the ERG b wave) was significant affected prior to any observable dysfunction in RGCs. Using patch-clamp recording, we also show that the rod, but not cone bipolar cell transduction current was diminished. These results suggest that examination of RBCs, in particular the ERG b wave, could potentially be an early diagnostic approach for glaucoma patients.

In our study, PKCα expression in RBCs decreased significantly following vitreal administration of NMDA. In addition, the CPPG-evoked transduction response, also diminished in response to NMDA treatment, was partially rescued by the PKCα activator PMA, whereas PKCα inhibitor GÖ6976 incubation completely blocked the RBCs transduction response. Consistently, the PKCα activator PMA treatment also rescued the expression of PKCα as shown by immunofluorescence staining, supporting the idea that NMDA treatment impairs RBC function through negatively regulating the expression of PKCα.

PKCα plays an important role in the modulation of TRPM1 channel of RBCs^10–12^, a critical transduction channel in RBCs. In PKCα knockout mice, the kinetics of the ERG b-wave was significantly changed with no obvious change in the amplitude^10^; the deletion of PKCα changes the scotopic ERG, but has no significantly effect on photonic ERG^12^.

The molecular mechanisms that control the stability of PKCα remain an important question. Activation-induced downregulation of PKC has been proposed in a recent study^41^. We observed NMDA-induced PKCα degradation in RBCs; one possible explanation for this is that phosphorylated NR2B is a substrate for PKCα^42^. A positive feedback loop in which NMDAR mediated CaMKII phosphorylation of NMDAR leads to increased Ca^2+^ influx and RGC excitotoxicity has been proposed^43^.

On the basis of the findings reported here, we propose a model for the interplay among PKCα, NMDARs, CaMKII, and PICK1 (Supplementary Fig. 4). PKCα activation is mediated through association with intracellular calcium, perhaps entering through NMDA receptors, resulting in an increase of its binding affinity to PICK1^15,44^. PICK1 is an anchor protein binding to PKCα and glutamate receptor through its PDZ domain in its N-terminal region^45,46^. Importantly, our results suggest that PICK1 is critical for the PKCα degradation in RBCs. The PKCα protein as well as rod bipolar cell function remained stable in NMDA-treated PICK1 knockout mice. We speculate that PICK1 protein serves as an anchor protein to promote association of PKCα with NR2B, as our results revealed that only the phosphorylation form of NR2B could induce PKCα degradation. In conclusion, the degradation of PKCα require the assistance of PICK1 and the phosphorylation of NR2B.

Our findings place PKCα-mediated RBCs degeneration in the early progress of NMDA-Induced retinal excitotoxicity, and suggest a molecular pathway for the regulation of PKCα in RBCs. Elevated IOP has been shown previously to decrease the efficiency of signal transmission from RBCs to AII AC, and subsequently the loss of sensitivity of downstream On RGCs^47^. Based on the present results, we propose that the decrease in synaptic efficiency occurs even earlier in the rod pathway, at the synapse between rods and RBCs. This work highlights the need to understand animal models of retinal disease in the broader context of the retinal circuits, and serves as a reminder that ganglion cells are not the only cell in the retina that is vulnerable.

## Materials and methods

### Animals

Male C57BL/6J mice (https://www.jax.org/strain/000664) (3–5 weeks) were obtained from Wuhan University Laboratory Animal Center and were kept under air-conditioned barrier system (light/dark cycle 12 h/12 h; room temperature, 23 ± 2 °C). PICK1^−/−^ mice (https://www.jax.org/strain/008891) of either sex were obtained from Jun Xia (Hong Kong University of Science of Technology, Hong Kong, China). The animal sample size in the article was chosen to ensure adequate power to detect a pre-specified effect size. The eyes of the mice were normal with no microphthalmia and anophthalmos, with clear cornea and lens, normal anterior chamber volume, normal intraocular pressure and sensitive reflection to light. The eyes would be excluded if they were infected or hemorrhaged after the operation. The mice were randomly assigned to interventions. The experimental procedures described were conducted in accordance with NIH guidelines for all animal experimentation.

### Intravitreal injection

Mice were anesthetized with an i.p. injection of 1% pentobarbital sodium (35 mg/kg). Intravitreal injection was performed with a 30-gauge needle connected to a 5 μl microsyringe (Hamilton, American). In the NMDA groups, NMDA (3 μl of 10 mM NMDA in saline solution, Sigma, 684-93-5) was injected into the vitreous cavity of the left eye and same volume of saline was administered into the fellow eye, which served as a control. The tip of the needle was inserted 1 mm behind the corneal limbus. Intravitreal injection was performed as previously described^48–50^. Drugs used in this experiment were KN93 (1 nM, Calbiochem, 422708), PMA (100 μM, Sigma, P1585) and MG132 (20 μg/ml, Sigma, M8699). For the MG132 + NMDA group, a mixture of 20 μg/ml MG132 and 10 mM NMDA was injected into the vitreous cavity of the left eye and saline into the right eyes as the control. For the ONX0912 + NMDA group, intravitreal injection of NMDA was performed on the 4th day of a five-day intragastric administration of ONX0912 (30 mg/kg, Onyx Pharmaceuticals, 935888-69-0).

### Electroretinogram (ERG)

Mice were dark adapted for 12 h before ERG recording, anesthetized with 1% pentobarbital sodium (35 mg/kg) and then injected with NMDA (3 μl of 0.05 mM solution) for the corresponding time under dim red light. The pupils were dilated by topical application of phenylephrine HCl (0.5%) and tropicamide (0.5%), and the eyes were lubricated and hydrated with 1% sodium hyaluronate. The ERG was recorded with a gold plated wire loop contacting the corneal surface as the active electrode (company). Stainless steel needle electrodes were inserted into the skin between the two ears and into the tail, serving as reference and ground leads, respectively. For scotopic ERG, responses to white flashes of 10^–3^ cds/m^2^, 10^–2^ cds/m^2^, 0.1 cds/m^2^, 1.0 cds/m^2^ and 3.0 cds/m^2^, and 10.0 cds/m^2^ were recorded. For photopic ERG, mice were light adapted for 5 min with green background (25 cds/m^2^), and photopic responses to green flashes of 10.0 cds/m^2^ were recorded. Responses were low passing filtered (50 Hz cutoff). For photopic negative responses (PhNR), the amplitude of the negative peak following the b-wave (the first positive peak in the ERG trace) was measured relative to the baseline.

### Retinal slice preparation

Retina slices were prepared from male wild-type 3–5 weeks old C57BL/6 mice in accordance with the NIH guidelines for animal experimentation. After euthanization, both eyes were removed and immersed in oxygenated Ames’ medium (Sigma-Aldrich, A1420) at room temperature (24 °C). Whole retinas were isolated and placed on a 0.65 μm cellulosed acetate/nitrate membrane filter (Millipore, USA) that was secured with vacuum grease to a glass slide adjacent to the recording chamber. Then slices were separated to a thickness of 150 μm using a tissue slicer (Stoelting, USA), and transferred to the recording chamber while remaining submerged, and viewed through an Olympus BX51WI microscope equipped with a water-immersion ×40 objective and differential interference contrast optics (DIC). Slices were perfused with Ames’ medium bubbled with 95% CO_2_ and 5% O_2_ at a rate of 4–7 ml/min.

### Electrophysiological recording and solution

All recording were obtained with patch-clamp amplifier (HEKA Elektronik, EPC 10) and had input and series of resistances of ~1 GΩ and 5–20 MΩ, respectively. Patch electrodes were fabricated from borosilicate glass using a two-stage vertical puller (Narishige, PC-10) and fire-polished to resistances of 5–7 MΩ. The patch pipette solution was composed of (in mM): 145 CsCl, 10 HEPES, 0.5 EGTA, 4 ATP, 1 GTP, adjusted to pH 7.3 with CsOH. L-AP4 (4 μM), an mGluR6 receptor agonist, was also included in the bath. The metabotropic receptor antagonist CPPG (100 μM), or AMPA receptor agonist AMPA (100 μM) were applied to the different bipolar cells dendrites from a pipette using a positive pressure (2–4 psi) with a computer-controlled solenoid valve (Picospritzer, USA). All chemicals were purchased from Sigma-Aldrich or Tocris Bioscience. Data acquisition and analysis were performed with Patchmaster and Igor Pro (Wave-metrics, USA). Data were filtered at 2 kHz and digitized at 5 kHz.

### Fundus imaging and angiography

Mice eyes were dilated with a mixed ophthalmic solution containing 0.5% atropine sulfate (Compound Tropicamide Eye Drops). The fundus was photographed with a Micron IV (Phoenix Research Lab, Pleasanton, CA, USA) fundus camera for animals. Photographs were taken with Micron IV containing a barrier filter for fluorescein angiography and processed for Photoshop for digital images.

### Western immunoblotting

Western blotting was processed as described previously^51^. For protein extraction, eyes were hemisected, and retinas were transferred immediately to a homogenizer preloaded with RIPA lysis buffer (50 mM Tris-HCl, 150 mM NaCl, 1% NP-40, 0.1% SDS, 0.5% sodium deoxycholate, 1 mM EDTA, 1 mM PMSF) containing a protease inhibitor cocktail. Total protein content was measured with a BCA Protein Assay kit, according to the manufacturer’s recommendations. Proteins (10 mg/lane) were subjected to 12% SDS-PAGE and electroblotted to nitro-cellulose membranes. The membrane was blocked with 5% non-fat milk in Tris-buffered saline (TBS, pH = 7.3) containing 20 mM Tris-HCl, 0.1% Tween 20, 137 mM NaCl for 2 h at room temperature, then the samples were incubated with primary antibody diluted in the blocking buffer overnight at 4 °C. The primary antibodies used were a rat antibody against NMDA receptor subunit 2B (NR2B, 1:1000, Millipore, AB1557), a rabbit antibody against phospho-NR2B (Ser1303) (p-NR2B, 1:1000, Millipore, 07-398), a rabbit antibody against PKCα (1:1000, Sigma, P4334) and a mouse monoclonal antibody against GAPDH (1:10,000, Abbkine, #A01020). The blots were washed completely with TBST for 5 times (5 min/time) and then incubated with HRP-conjugated goat anti-mouse or goat anti-rabbit IgG (1:1000, PTGLab) for 2 h at room temperature. The immunoreactive bands were developed with enhanced chemiluminescence and observed by photographic film. No band was shown when the first antibody was omitted. To estimate the molecular weight (MW) of proteins, a prestained marker (Fermentas, Maryland, USA) was used. Protein levels were analyzed using densitometry via Photoshop (Adobe, USA).

### Immunofluorescence staining

The procedure used had been described elsewhere^51^. The retinal sections were initially blocked with 5% bovine serum albumin in PBS for 2 h at room temperature. Then the section was incubated with primary antibody in 5% bovine serum albumin overnight or 3 days at 4 °C. After extensive washing with PBS for 5 min × 5 times, the sections were incubated with secondary antibody. Nuclear counterstaining was labeled with 4,6-diamidine 2-phenylindoledihydrochloride (DAPI). The slides were mounted using anti-fade mounting media. All antibodies used here were obtained commercially and diluted according to the manufacturer’s instruction (the dilution was also confirmed in our laboratory). The primary antibodies used for labeling were as followed: A goat polyclonal antibody against PKCα (1:1000, Santa Cruz, sc-208) and rabbit polyclonal antibody against PKCα (1:1000, Sigma, P4334) were used to label rod bipolar cells (Rod-BCs). Also a goat polyclonal antibody against PCP2 (1:200, Santa Cruz, sc-49072) was used to label Rod bipolar cells. Antibodies were used to label NMDA receptors subunits: Mouse monoclonal antibody against NR1 (1:200, BD Pharmingen, 556308), rabbit antibody against NR2B (1:200, Millipore, AB1557). The secondary antibodies used in the research were as follow: Alexa Fluor 594 affinipure donkey anti-goat IgG (1:500, Jackson Immuno Research, 705-585-003), Alexa Fluor 594 affinipure donkey anti-rabbit IgG (1:500, Jackson Immuno Research, 711-585-152), Alexa Fluor 488 affinipure donkey anti-rabbit IgG (1:500, Jackson Immuno Research, 711-545-152), Alexa Fluor 488 affinipure donkey anti-mouse IgG (1:500, Jackson Immuno Research, 715-545-150). Images of immunohistochemical staining were collected on fluorescence microscope (BX53, Olympus, Tokyo, Japan) or with confocal fluorescence microscopy (Olympus FV1200).

### Retinal flat-mount immunofluorescence

For whole-mount retina staining, the anterior segment of the eye and vitreous humor were removed, enucleated eyes were incubated in 4% paraformaldehyde for 1 h. The retina was dissected from the sclera and flattened on a glass slide with four incisions. The retinas were then blocked in buffer containing 5% bovine serum albumin (BSA) and 0.2% Triton X-100 in PBS at RT for 1 h. Then the retinas were incubated overnight with Griffonia simplicifolia isolectin B4 conjugated to Alexa Fluor 594 (1:200, Invitrogen, 121413) or primary antibody followed by incubation with Cy3-conjugated secondary antibodies at RT for 3 h. Retinas were placed in anti-fade mounting medium, and images were captured using a fluorescence microscope.

### Cell culture and lipofectamine transfection

The HEK293T human embryonic kidney cell line (ATCC, CRL-3216) was cultured in DMEM supplemented with 10% fetal bovine serum (FBS) and 1% glutamine Pen-Strep solution at 37 °C and 5% CO_2_. Lipofectamine transfections were performed by co-transfection of NR2B-WT-Flag (or NR2B-S2E-Flag/NR2B-S2A-Flag), PKCα-HA, PICK1-Myc and GFP plasmids. The medium (on 85–90% confluent HEK293 cells in three plates of 10-cm diameter per trial) was changed to DMEM-10% FBS without 1% glutamine Pen-Strep solution 2 h before transfection. The ratios of the three plasmids (μg) to lipofectamine 2000 (μl) (Invitrogen, 1854316) were 1:1. Plasmids (NR2B-WT or NR2B-S2A or NR2B-S2E, PKCα, PICK1, and GFP plasmids, at the same ratio: 10 μg plasmid: 40 μl lipofectamine 2000) were conjugated to lipofectamine and added drop wise to plates. The cultures were further incubated. HEK293 cells were harvested 36 h after transfection for further analysis.

### Co-Immunoprecipitation

The HEK 293 T cells were washed twice with cold PBS and lysed with lysis buffer (50 mM Tris-HCl pH 7.4, 1% Triton X-100, 1 mM EDTA, 1 mM leupeptin, 1 mM phenylmethylsulfonyl fluoride, 10 mM NaF and 1 mM Na_3_VO_4_) 36 h after transfection with plasmids. The supernatant was obtained by centrifugation (12,000 × rpm, 4 °C, 10 min) and then mixed with target antibodies from mouse IgG (Anti-DDDDK-tag mAb, MBL, M185-3L; Anti-HA-tag mAb, MBL, M180-3; Anti-Myc-tag mAb, MBL, M192-3) correspondingly or matched control IgG (Santa Cruz Biotechnology, L1216) as a negative control for 6 h at 4 °C with rotation. Fresh protein A/G agarose beads (Santa Cruz Biotechnology, L0516) was then added, followed by an overnight incubation at 4 °C with rocking. Immunoprecipitates were centrifuged at 1000 rpm for 2 min at 4 °C. The supernatant was discarded and the pellet was washed four times with lysis buffer and then resuspended with the same volume of 1× SDS buffer at 100 °C for 10 min. Separated the samples by SDS-polyacrylamide gel electrophoresis (SDS-PAGE) with target antibodies from rabbit IgG (Anti-DDDDK-tag mAb, MBL, 025; Anti-HA-tag mAb, MBL, 065; Anti-Myc-tag mAb, MBL, 055).

### Animal model of acute ocular hypertension (AOH)

The animals were anesthetized with an i.p. injection of 1% pentobarbital sodium (35 mg/kg). The IOP was increased to almost 100 mmHg detected by iCare rebound tonometer after 1 h, by using a 500 ml sterile physiological saline elevated to a height of 1.5 m which connected to a 32-gauge needle placed in the anterior chamber of the left eyes. Sham procedure eyes were treated similarly but without the elevation of the sterile physiological saline; hence, the normal IOP was maintained.

### Optic nerve crush

We performed optic nerve crush (ONC) on all of the left eyes, whereas right eyes served as contra-lateral healthy controls. An incision was made at the superolateral aspect of the conjunctivae. Through this incision, a blunt dissection of the conjunctiva was made with forceps towards the back of the eye to expose the retrobulbar optic nerve. The optic nerve was crushed with cross-action Dumont tweezers for 5 s, ~2 mm posterior to the globe. Special care was taken to protect surrounding blood vessels. After the surgery, antibiotic ophthalmic ointment was applied to avoid infection and the animal recovered on a warming pad.

### Statistics

Investigators were blinded to the group allocation during the experiment and/or when assessing the outcome, state the extent of blinding. Current records were initially analyzed by the Patch Master and Igor to assess whole-cell current amplitude and kinetics. Data were analyzed with GraphPad Prism 7.0 and expressed as the mean ± S.E.M. *t*-tests was performed to assess significance of differences, and *p*-values less than 0.05 were considered to be significant (^*^P < 0.05, ^**^P < 0.01, ^***^P < 0.001, N.S.: no significance).

## Supplementary information

Supplementary figure legends

Checklist

Supplementary figure 1

Supplementary figure 2

Supplementary figure 3

Supplementary figure 4
