# Supplementary material for: Rod bipolar cells dysfunction occurs before ganglion cells loss in excitotoxin-damaged mouse retina
Source: Cell Death Dis. 2019 Dec 2;10(12):905. doi: 10.1038/s41419-019-2140-x (PMC6885518; doi:10.1038/s41419-019-2140-x)
Supplement: Supplementary file 1 — Supplementary figure legends [file 41419_2019_2140_MOESM1_ESM.docx]

**Supplementary Figure legends**

**Supplementary Figure 1: RGCs were sensitive to NMDA treatment in a time-depended manner.**

(A) HE staining of mouse retina treated with vehicle or NMDA. RGCs decreased time-dependently with NMDA treatment. (B) Retina of optic nerve crush model stained with 2% toluidine blue, revealing axonal damage as *. (C) Brn3a^+^ RGCs decreased time-dependently with NMDA treatment in flat-mounted retinas (Scale bar = 50 μm).

**Supplementary Figure 2: NMDA treatment induced NMDA receptor subunits expression changes.**

NR1, NR2B and NR2D expression increased after NMDA treatment in a time-depended manner, meanwhile with no change of NR2A, NR2C subunits (Scale bar = 50 μm).

**Supplementary Figure 3:** **NMDA induced PKCα degradation in RBCs dendrites was mediated by synaptic NMDA receptor.**

NMDA treatment caused RGCs death and decrease expression of PKCα (A, B), NMDA receptor channel blocker MK801 (C) but not memantine, a parasynaptic NMDA receptor antagonist(D), could reverse PKCα expression (Scale bar = 50 μm). PKCα intensity analysis in all groups shown in (E). Data represent the mean ± s.e.m. ***P  ≤ 0.001, two-way ANOVA.

**Supplementary Figure 4: Schematic diagram depicting the proposed interaction of PKCα, PICK1 and NR2B.**

The influx of Ca^2+^ activates CaMKII, leading to phosphorylation of NR2B. Phosphorylated NR2B can now interact with PKCα through PICK1 protein, and ultimately induces PKCα degradation by the ubiquitin-proteasome system. KN93: inhibitor of CaMKII; ONX and MG: inhibitors of proteasome; Gö6976: inhibitor of PKCα.
