# Supplementary figures and images for: Rod bipolar cells dysfunction occurs before ganglion cells loss in excitotoxin-damaged mouse retina
Source: Cell Death Dis. 2019 Dec 2;10(12):905. doi: 10.1038/s41419-019-2140-x (PMC6885518; doi:10.1038/s41419-019-2140-x)

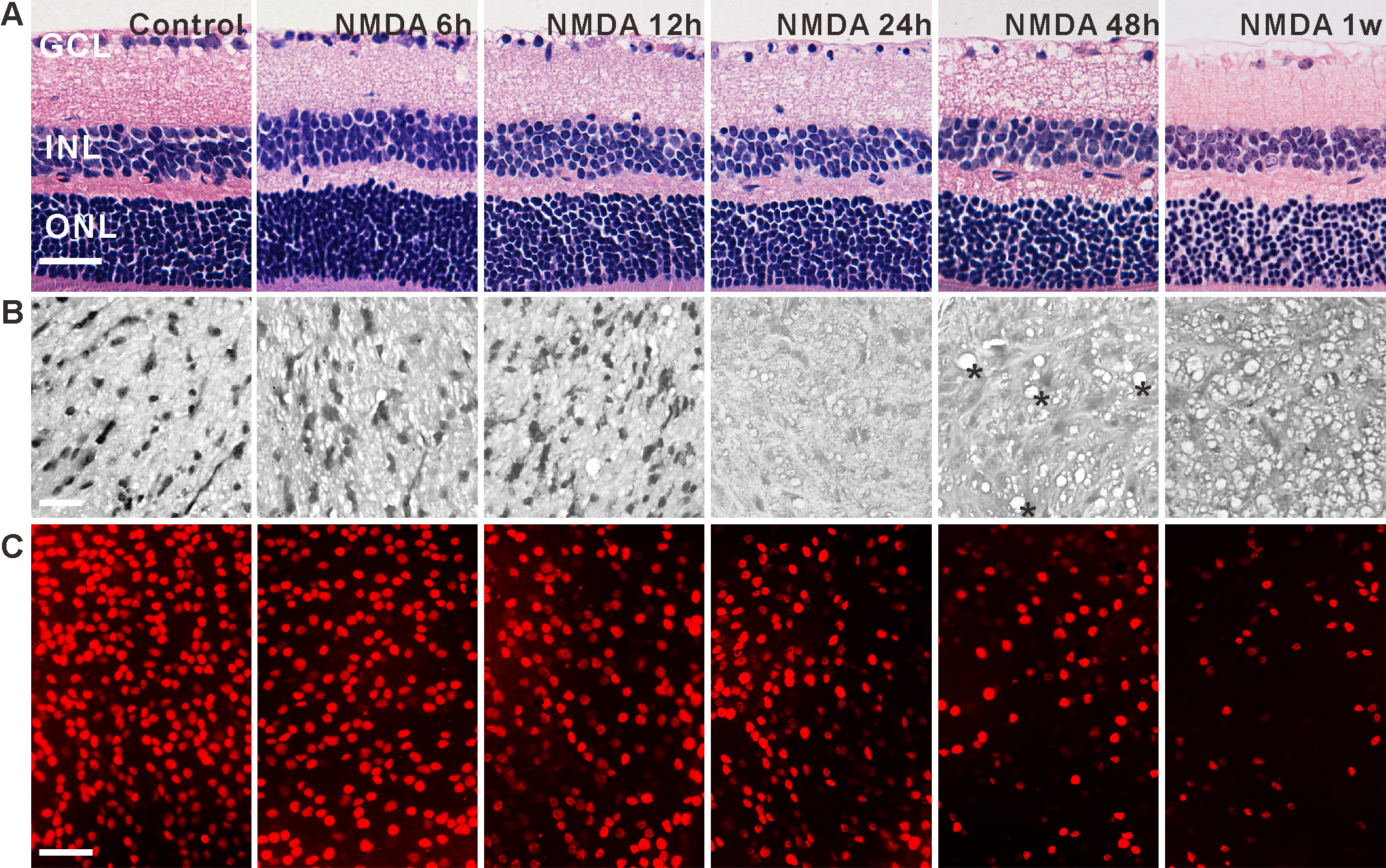

Supplement: Supplementary file 3 — Supplementary figure 1 [file 41419_2019_2140_MOESM3_ESM.png]

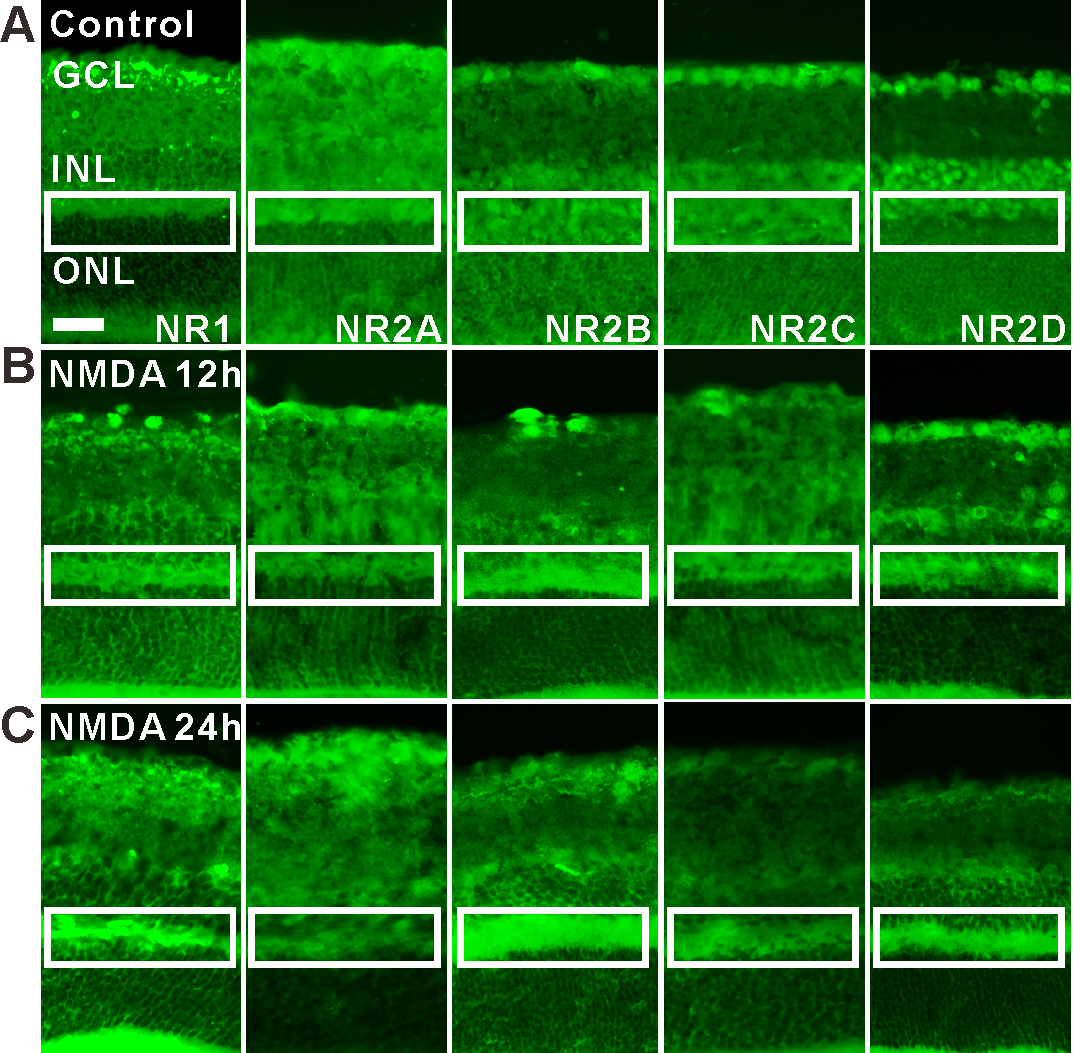

Supplement: Supplementary file 4 — Supplementary figure 2 [file 41419_2019_2140_MOESM4_ESM.png]

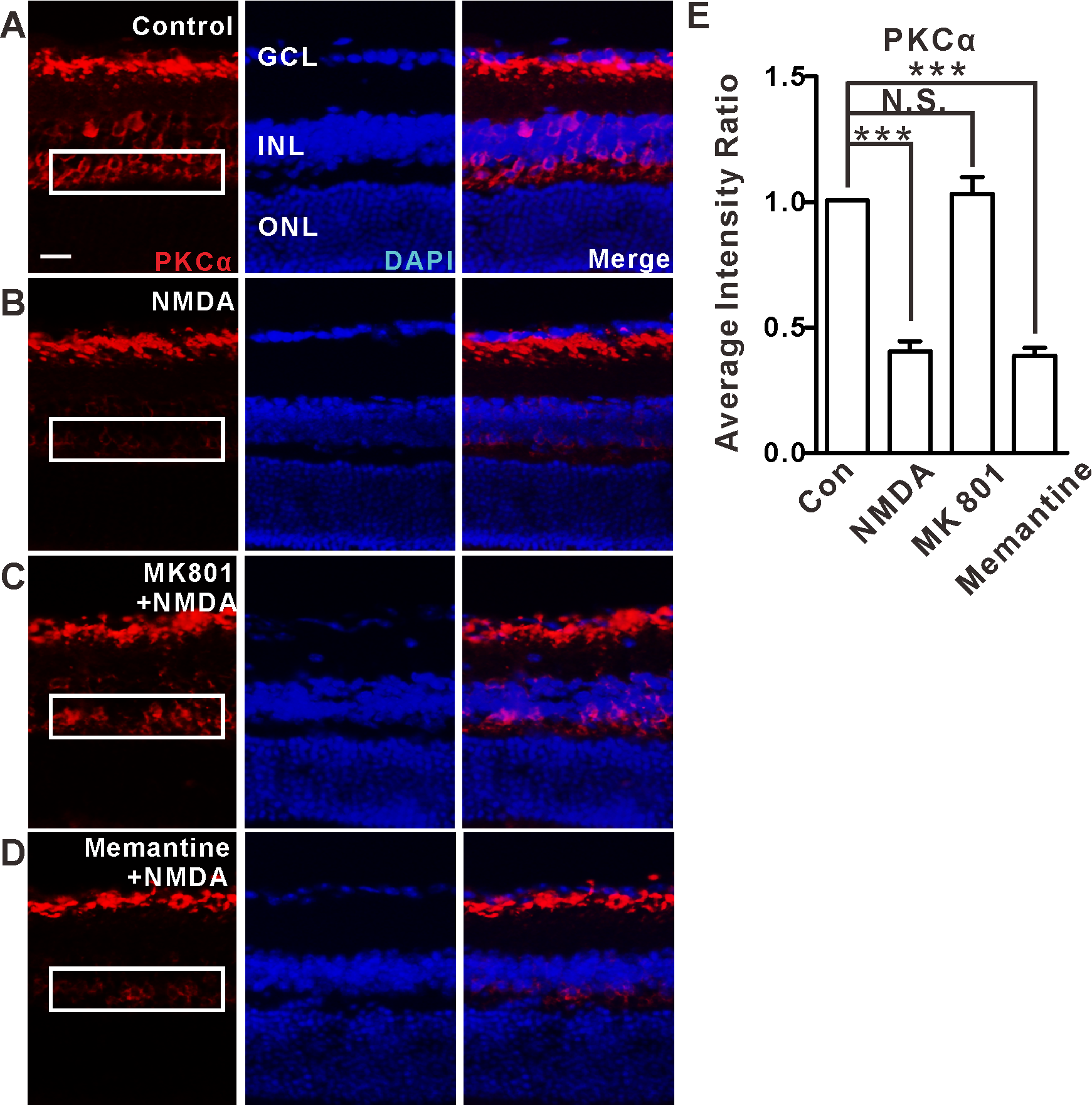

Supplement: Supplementary file 5 — Supplementary figure 3 [file 41419_2019_2140_MOESM5_ESM.png]

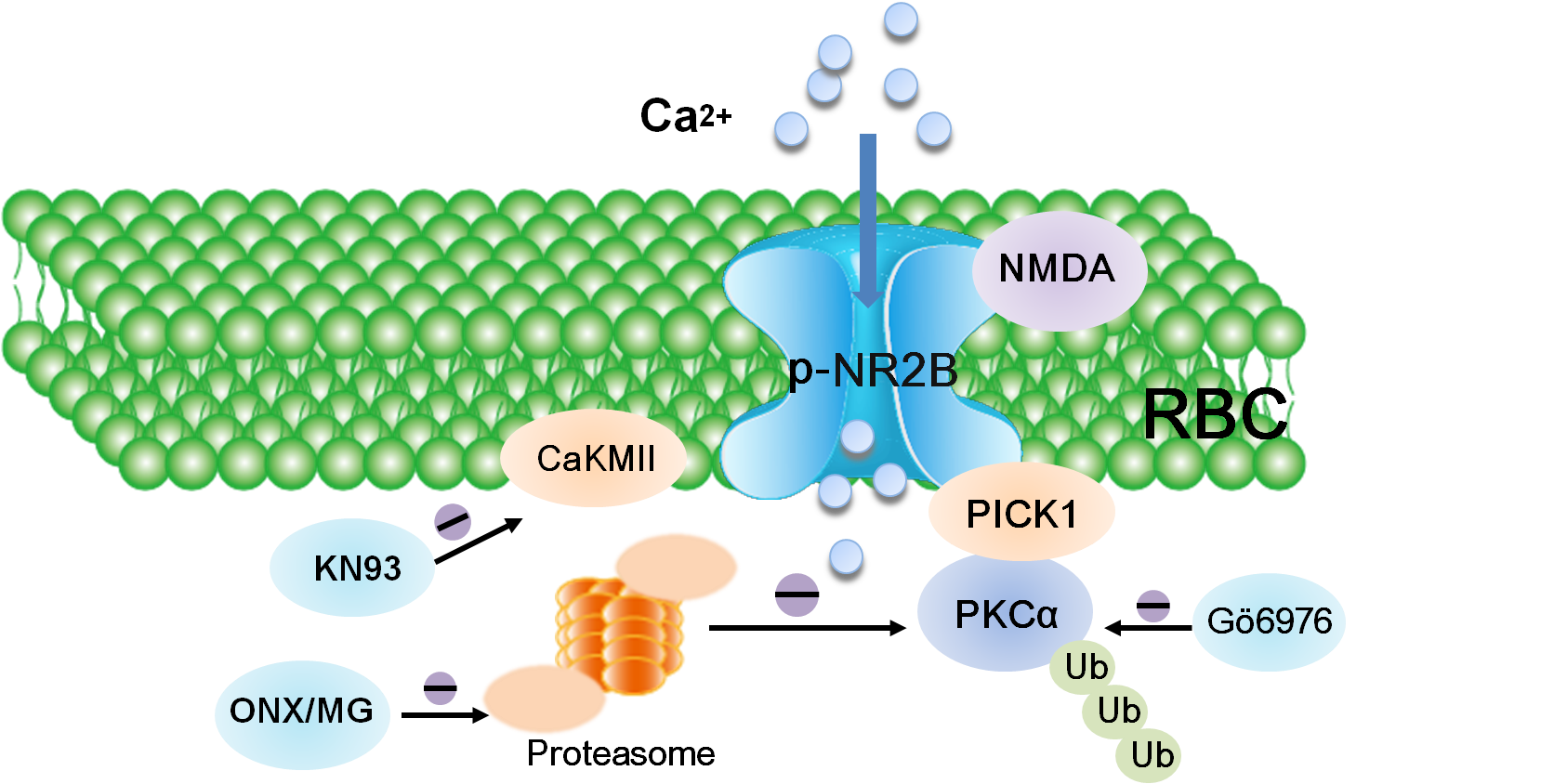

Supplement: Supplementary file 6 — Supplementary figure 4 [file 41419_2019_2140_MOESM6_ESM.tif]
